# Evaluation of NCAM1, NRXN1, NLGN4, N-CADHERİN Levels in Peripheral Circulation of Children with Autism Spectrum Disorder and Their Siblings

**DOI:** 10.1192/j.eurpsy.2025.653

**Published:** 2025-08-26

**Authors:** A. T. Hira Selen, N. Uzun, I. Kılınc, A. O. Kılıc

**Affiliations:** 1 Child and Adolescent Psychiatry; 2 Clinical Biochemistry; 3 Department of Pediatrics, Necmettin Erbakan University, Konya, Türkiye

## Abstract

**Introduction:**

Autism spectrum disorder (ASD) is linked to synaptic function and plasticity, and cell adhesion molecules play an important role in these processes. Dysfunction of CAMs disrupts synaptic activity and plasticity processes.

**Objectives:**

This study aims to investigate NCAM1, NRXN1, NLGN4, and N-cadherin levels in the peripheral circulation of individuals with ASD, their siblings, and healthy controls without any psychiatric diagnosis. We also investigate how these biochemical parameters affect autism severity, behavioral problems, and autistic traits in siblings.

**Methods:**

The patient group of the study consisted of 41 children aged between 18-72 months who were diagnosed with ASD according to DSM-5 diagnostic criteria and 41 healthy siblings aged between 24-72 months of the child diagnosed with ASD (Control Group 1). We assessed the severity of ASD symptoms in participants diagnosed with ASD using the Childhood Autism Rating Scale and the Autism Behavior Checklist. Control Group 2 consisted of 41 children aged between 18-72 months who did not have ASD or any other psychiatric disorder or physical illness (Control Group 2). The parents of the patient group completed the Autism Spectrum Screening Scale for the sibling of the individual diagnosed with ASD. We determined serum NCAM1, NRXN1, NLGN4, and N-Cadherin levels in peripheral blood samples by ELISA.

**Results:**

There was no statistically significant difference in NCAM1, NRXN1, NLGN4, and N-Cadherin levels between all three study groups. There was a statistically significant positive correlation between NCAM1 and NRXN1 levels in the ASD group.

**Conclusions:**

In this study, we investigated the serum levels of NCAM1, NRXN1, NLGN4, and N-Cadherin, which are cell adhesion molecules potentially associated with ASD etiology. Although the proteins we investigated were expressed differently between the groups, we did not find any statistically significant difference between ASD patients, healthy siblings, and healthy groups. More research is needed in this area.

**Disclosure of Interest:**

None Declared

